# Multiple Mainlobe Interferences Suppression Based on Eigen-Subspace and Eigen-Oblique Projection

**DOI:** 10.3390/s22218494

**Published:** 2022-11-04

**Authors:** Yunhao Ji, Yaobing Lu, Shan Wei, Zigeng Li

**Affiliations:** Beijing Institute of Radio Measurement, Beijing 100854, China

**Keywords:** mainlobe interference suppression, eigen-subspace, adaptive beamforming, eigen-oblique

## Abstract

When the desired signal and multiple mainlobe interferences coexist in the received data, the performance of the current mainlobe interference suppression algorithms is severely challenged. This paper proposes a multiple mainlobe interference suppression method based on eigen-subspace and eigen-oblique projection to solve this problem. First, use the spatial spectrum algorithm to calculate interference power and direction. Next, reconstruct the eigen-subspace to accurately calculate the interference eigenvector, then generate the eigen-oblique projection matrix to suppress mainlobe interference and output the desired signal without distortion. Finally, the adaptive weight vector is calculated to suppress sidelobe interference. Through the above steps, the proposed method solves the problem that the mainlobe interference eigenvector is difficult to select, caused by the desired signal and the mismatch of the mainlobe interference steering vector and its eigenvector. The simulation result proves that our method could suppress interference more successfully than the former methods.

## 1. Introduction

Adaptive beamforming is a key research area in array signal processing. It has numerous uses in radar [1], sonar, navigation, electronic warfare [2], wireless communication, and so on. In modern electronic warfare, intentional interference by the enemy makes it difficult for radar to detect targets. Sidelobe interference can be successfully suppressed using conventional adaptive beamformers. However, when mainlobe interference exists in the received data, a null will be formed in the mainlobe, resulting in the distortion of the mainlobe and reducing the output signal to interference plus noise ratio (SINR). Therefore, when the radar is fighting against mainlobe interference, conventional adaptive beamforming is no longer applicable [3].

To solve this problem, references [4,5] uses large aperture auxiliary arrays to suppress mainlobe interference, but such methods require a large amount of space for antenna array layout. In [6,7], the antenna spatial polarization characteristics are used to suppress mainlobe interference in the polarization domain, but such methods require radar to have the capability of polarimetric measuring. The blind source separation (BSS) method is proposed to estimate mainlobe interference waveform and signal steering vector in [8,9,10]; the real-time canonical correlation analysis (RCCA) algorithm proposed by Bhowmik B on the basis of BSS can process data in real-time [11]. However, the algorithm based on BSS is difficult to obtain the direction of arrival (DOA) of the target signal while separating the target signal from the interference [12]. The blocking matrix preprocessing (BMP) method is proposed in [13], which can achieve good performance under the condition of knowing the interference direction. However, it will reduce the array’s degrees of freedom.

The eigenprojection matrix preprocessing (EMP) method proposed in [13] can achieve good mainlobe interference suppression without reducing array degrees of freedom. Scholars have carried out a series of studies based on this method. The EMP-CMR method is proposed in [2] based on [13] and eliminated mainlobe offset by reconstructing the received data’s covariance matrix. The EMP-SC method proposed in [14] can effectively suppress multiple mainlobe interferences; Reference [15] constrains the sidelobe level based on [14], which reduces the sidelobe level while ensuring the interference suppression capability. However, all the above three methods require that there is no desired signal in the received data. The Capon spatial–spectral estimation is used in [16] to reconstruct the interference plus noise covariance matrix (INCM). However, the eigen-oblique projection changes the eigenvector of sidelobe interference so that the processed sidelobe interference cannot be suppressed by the adaptive weight vector. Reference [17] corrects the sidelobe interference plus noise covariance matrix (SINCM) based on [16], but in the process of correction, the noise is reconstructed into color noise, which reduces the suppression performance of sidelobe interference. Reference [18] reduced the loss of the desired signal by eigen-oblique projection. However, when there are multiple mainlobe interferences, the accurate mainlobe interference eigenvector cannot be obtained, which reduces the suppression performance of mainlobe interference. In [19], the iterative adaptive algorithm (IAA) is proposed to estimate mainlobe interference DOA. This method can effectively suppress coherent interference, but the Frobenius norm has to be calculated in each iteration, which leads to high algorithm complexity. Reference [20] estimates the power of sources by compressive sensing (CS) and reconstructs the INCM, but there is a mismatch in the estimation of the interference eigenvector. The matrix filter is proposed in [21] to estimate the number of mainlobe interferences, but the selection of the threshold is difficult to determine. Reference [22] constructs the blocking matrix by inverting INCM. This method improves the robustness of desired signal mismatch but can only suppress a single mainlobe interference.

The above methods all have problems in the suppression of mainlobe interference. Therefore, this paper proposes a multiple mainlobe interference suppression method based on eigen-subspace and eigen-oblique projection. We improve the reconstruction method of eigen-subspace. It can not only obtain a more accurate interference eigenvector but also improve the accuracy of SINCM reconstruction. Based on the traditional eigen-projection method, we further reduce the loss of the desired signal and preserve the spatial characteristics of sidelobe interference by eigen-oblique projection. It improves the performance of sidelobe interference suppression. The proposed method has the following advantages:

1. This method has higher interference suppression ratio (ISR) when the desired signal and multiple mainlobe interferences coexist in the received data; that is, the interference suppression capability is better than other methods;
2. This method has low complexity and a fast convergence rate and is able to minimize the desired signal loss while suppressing mainlobe interference.

The rest of this paper is arranged as follows. In Section 2, the array signal model in a multiple mainlobe interference scenario is introduced. In Section 3, the mainlobe interference suppression method based on eigen-subspace and eigen-oblique projection is proposed. The numerical results are presented and analyzed in Section 4. Finally, the conclusion is given in Section 5.

## 2. Signal Model

Suppose a uniform linear array (ULA) with N omnidirectional antennas; the received signal satisfies far field narrowband assumption. The $k^{th}$ received data sample of ULA is as follows:

(1) $$\begin{array}{cc} x\left(k\right) & =x_{d}\left(k\right)+x_{Mi}\left(k\right)+x_{Si}\left(k\right)+x_{n}\left(k\right)\\ & =a\left(\theta_{0}\right)s_{0}\left(k\right)+\sum_{m=1}^{M}a\left(\theta_{m}\right)s_{m}\left(k\right)+\sum_{m=M+1}^{M+S}a\left(\theta_{m}\right)s_{m}\left(k\right)+n\left(k\right) \end{array}$$

where $x_{d}\left(k\right)=a\left(\theta_{0}\right)s_{0}\left(k\right)$, $x_{Mi}\left(k\right)=\sum_{m=1}^{M}a\left(\theta_{m}\right)s_{m}\left(k\right)$, $x_{Si}\left(k\right)=\sum_{m=M+1}^{M+S}a\left(\theta_{m}\right)s_{m}\left(k\right)$ represent the desired signal, mainlobe interference, sidelobe interference, respectively. M represents the number of mainlobe interferences. S represents the number of sidelobe interferences. $x_{n}\left(k\right)=n\left(k\right)$ represents independent zero-mean additive white Gaussian noise. $a\left(\theta_{0}\right)$ represents desired signal steering vector. $a\left(\theta_{m}\right)$ represent interference steering vector. The signal steering vector with direction θ is:

(2) $$a\left(\theta \right)={\left[1,e^{-j\frac{2\pi d}{\lambda }\sin\theta },\ldots ,e^{-j\frac{2\pi \left(N-1\right)d}{\lambda }\sin\theta }\right]}^{T}$$

where ${\left(\;\;\right)}^{T}$ represents the transpose of matrix, λ represents the signal wavelength.

After eigen-decomposition, the received data covariance matrix is as follows:

(3) $$\begin{array}{cc} R_{x} & =E\left[x\left(k\right)x^{H}\left(k\right)\right]=\sum_{i=1}^{N}{\tilde{\lambda }}_{i}{\tilde{u}}_{i}{\tilde{u}}_{i}{}^{H}\\ & ={\tilde{U}}_{s}{\tilde{\Lambda }}_{s}{\tilde{U}}_{s}{}^{H}+{\tilde{U}}_{n}{\tilde{\Lambda }}_{n}{\tilde{U}}_{n}{}^{H} \end{array}$$

where ${\tilde{\lambda }}_{1}>{\tilde{\lambda }}_{2}>\ldots >{\tilde{\lambda }}_{N}$ represent the ordered eigenvalues, ${\tilde{u}}_{i}$ is corresponding eigenvector, $U_{s}$ is signal subspace consist of ${\tilde{u}}_{1},{\tilde{u}}_{2},\cdots {\tilde{u}}_{M+S+1}$, $U_{n}$ is noise subspace consist of ${\tilde{u}}_{M+S+1},{\tilde{u}}_{M+S+2},\cdots {\tilde{u}}_{N}$.

According to signal subspace theory, when there are multiple interferences in the mainlobe, if the spatial difference and intensity difference between them is small, then the mainlobe interference steering vector will be inconsistent with the corresponding eigenbeam’s eigenvector, and the steering vectors of mainlobe interferences cannot be approximated by eigenvectors.

The output data of array antenna is:

(4) $$y\left(k\right)=\sum_{i=1}^{N}w_{i}^{*}x_{i}\left(k\right)=w^{H}x\left(k\right)$$

where $w={\left[w_{1},w_{2},\ldots ,w_{N}\right]}^{T}$ is obtained by the minimum variance distortionless response (MVDR) beamformer, the design criteria is [23]:

(5) $$\begin{array}{l} \underset{w}{\min}\;\;w^{H}R_{i+n}w\\ st\;\;w^{H}a\left(\theta_{0}\right)=1 \end{array}$$

$R_{i+n}$ represents the interference plus noise covariance matrix (INCM) that cannot be obtained in practice. Replace $R_{i+n}$ with the sample covariance matrix $R_{x}$:

(6) $$R_{x}=\frac{1}{K}\sum_{k=1}^{K}x\left(k\right)x^{H}\left(k\right)$$

where K is snapshots number. Finally, the adaptive weight vector can be expressed as:

(7) $$W_{opt}=\frac{R_{x}^{-1}a\left(\theta_{0}\right)}{a^{H}\left(\theta_{0}\right)R_{x}^{-1}a\left(\theta_{0}\right)}$$

## 3. Proposed Method

**Figure 1:**
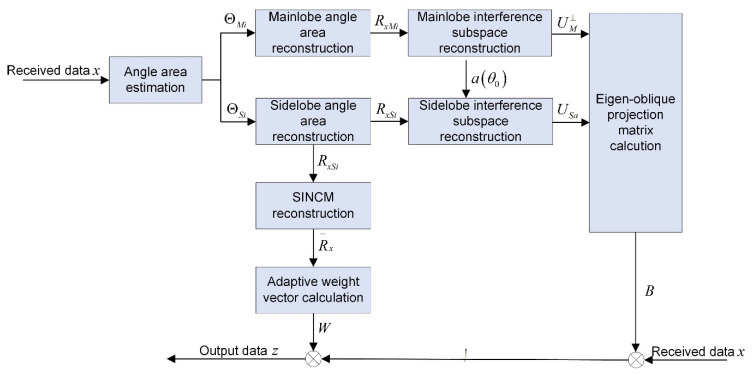
Processing diagram of the proposed method.

The proposed method will be introduced in detail in this section, and the processing diagram is shown in Figure 1.

### 3.1. The Construction of Eigen-Oblique Projection Matrix

In this section, we will introduce how to classify the interference, find the angle area where each interference is located, and then reconstruct the eigen-subspace to obtain the more accurate interference eigenvector to construct an eigen-oblique projection matrix. Finally, the data received by the projection matrix is preprocessed to reduce the loss of the desired signal while suppressing mainlobe interference.

The mainlobe width $M_{wid}$ calculation formula of ULA is as follows [24]:

(8) $$M_{wid}=2\arcsin\left(\frac{\lambda }{Nd}+\sin\theta_{0}\right)$$

where $\theta_{0}$ represents the desired signal direction, assuming that the desired signal receiving area is:

(9) $$\Theta_{0}=\left[\theta_{0}-\Delta \theta_{0},\theta_{0}+\Delta \theta_{0}\right]$$

Therefore, the incident angle area of mainlobe interference can be expressed as:

(10) $$\Theta_{m}=\left[\theta_{0}-\frac{M_{wid}}{2},\theta_{0}-\Delta \theta_{0}\right]\cup \left[\theta_{0}+\Delta \theta_{0},\theta_{0}+\frac{M_{wid}}{2}\right]$$

Calculate the signal power of each angle by using MUSIC spectrum estimation in $\Theta_{m}$ [25]:

(11) $$P_{Music}\left(\theta \right)=\frac{1}{a^{H}\left(\theta \right){\tilde{U}}_{n}{\tilde{U}}_{n}{}^{H}a\left(\theta \right)}$$

The area $\Theta_{m}$ is divided into grids, calculate the spatial spectral function $P_{Music}\left(\Theta_{m}\right)$ in this area according to Equation (12), and the mainlobe interference angle $\varphi_{Mi}\;\left(i=1,2,\ldots ,M\right)$ can be located by searching for the spectrum peak value. Therefore, the angle area where the single mainlobe interference exists is:

(12) $$\Theta_{Mi}=[\varphi_{Mi}-\frac{\delta }{2},\varphi_{Mi}+\frac{\delta }{2}]\;\;\;\;\;i=1,2,\ldots ,M$$

where δ represents the interval width for reconstructing the interference. The grid division will cause errors in the estimated mainlobe interference angle, the mainlobe interference steering vector is inconsistent with the corresponding steering vector. Therefore, it is necessary to perform the secondary integral reconstruction to obtain a more accurate mainlobe interference steering vector.

For each angle area of mainlobe interference, reconstruct INCM of this angle area respectively:

(13) $$R_{xMi}=\sum_{j=1}^{\Theta_{Mi}/\Delta \theta }\frac{a\left(\theta_{j}\right)a^{H}\left(\theta_{j}\right)}{a^{H}\left(\theta_{j}\right){\tilde{U}}_{n}{\tilde{U}}_{n}{}^{H}a\left(\theta_{j}\right)}\Delta \theta \;\;\;\;\;i=1,2,\ldots ,M$$

In this process, we adopt the MUSIC spectrum to estimate the source’s power and direction. When the interference interval is greater than a quarter of the main beam, the angle estimation result is relatively robust. The estimation accuracy of the interference angle directly affects the spatial characteristics of the eigenvectors after the reconstruction of INCM. Therefore, when the training data contains the desired signal, the method can handle up to two mainlobe interferences; when the training data does not contain the desired signal, the method can handle up to three mainlobe interferences. We adopt the amplitude of the MUSIC spectrum as the source power to reconstruct INCM. The estimation error of interference power mainly affects the eigenvalue corresponding to the interference. From this point of view, the influence of the interference power estimation error is small.

After eigen-decomposition, $R_{xMi}$ can be expressed as:

(14) $$R_{xMi}=\sum_{j=1}^{N}\lambda_{ij}u_{ij}u_{ij}{}^{H}$$

where $\lambda_{i1}>\lambda_{i2}>\ldots >\lambda_{iN}$ are the ordered eigenvalues, $u_{ij}$ is the corresponding eigenvector.

When the sources are incoherent, the steering vectors of the sources are stretched into the same space as the signal subspace, and the signal subspace is orthogonal to the noise subspace [25]. Therefore, for each $R_{xMi}$ containing only a single mainlobe interference, the signal subspace contains only a single eigenvector, which is linearly related to the main lobe interference steering vector of the angle area. Therefore, the mainlobe interference steering vector can be equivalently replaced by the eigenvector of its corresponding eigen-beamforming:

(15) $$P_{i}a\left(\theta_{i}\right)a^{H}\left(\theta_{i}\right)=\lambda_{i1}u_{i1}u_{i1}{}^{H}$$

Therefore, the eigenvector of the largest eigenvalue in each area is used to replace the corresponding mainlobe interference steering vector to reconstruct the mainlobe interference subspace:

(16) $$U_{M}=\left[u_{11},u_{21},\ldots ,u_{M1}\right]$$

The mainlobe interference subspace’s orthogonal complement space is as follows:

(17) $$U_{M}^{⊥}=I-U_{M}{\left(U_{M}{}^{H}U_{M}\right)}^{-1}U_{M}{}^{H}$$

The incident angle area of sidelobe interference can be expressed as:

(18) $$\Theta_{s}=\left[-90^{\circ },\theta_{0}-\frac{M_{wid}}{2}\right]\cup \left[\theta_{0}+\frac{M_{wid}}{2},90^{\circ }\right]$$

Calculate the signal power of each angle by using capon spectrum estimation in $\Theta_{s}$ [23]:

(19) $$P_{Capon}\left(\theta \right)=\frac{1}{a^{H}\left(\theta \right)R_{x}{}^{-1}a\left(\theta \right)}$$

The area $\Theta_{s}$ is divided into grids, calculate the spatial spectral function $P_{Capon}\left(\Theta_{s}\right)$ in this area according to Equation (19), and the sidelobe interference angle $\varphi_{Si}\;\left(i=1,2,\ldots ,S\right)$ can be located by searching for the spectrum peak value. Therefore, the angle area where the single sidelobe interference exists is:

(20) $$\Theta_{Si}=[\varphi_{Si}-\frac{\delta }{2},\varphi_{Si}-\frac{\delta }{2}]\;\;\;\;i=1,2,\ldots ,S$$

For each angle area of sidelobe interference, reconstruct INCM of the angle area respectively:

(21) $$R_{xSi}=\sum_{j=1}^{\Theta_{Si}/\Delta \theta }\frac{a\left(\theta_{j}\right)a^{H}\left(\theta_{j}\right)}{a^{H}\left(\theta_{j}\right)R_{x}{}^{-1}a\left(\theta_{j}\right)}\Delta \theta \;\;\;\;i=1,2,\ldots ,S$$

After eigen-decomposition, $R_{xSi}$ can be expressed as:

(22) $$R_{xSi}=\sum_{j=1}^{N}\gamma_{ij}v_{ij}v_{ij}{}^{H}$$

where $\gamma_{i1}>\gamma_{i2}>\ldots >\gamma_{iN}$ are the ordered eigenvalues, $v_{ij}$ represents the corresponding eigenvector.

Therefore, the largest eigenvalue’s eigenvector in each area is used to replace the corresponding sidelobe interference steering vector, and use it together with $a\left(\theta_{0}\right)$ to reconstruct the desired signal plus sidelobe interference subspace:

(23) $$U_{Sa}=\left[a\left(\theta_{0}\right),v_{11},v_{21},\ldots ,v_{S1}\right]$$

The eigen-oblique projection can reduce the desired signal loss while suppressing mainlobe interference [18]. It can be expressed as:

(24) $$B=U_{Sa}{\left(U_{Sa}{}^{H}U_{M}^{⊥}U_{Sa}\right)}^{-1}U_{Sa}{}^{H}U_{M}^{⊥}$$

The product of the projection matrix B and $U_{Sa}$ is $U_{Sa}$ itself, and the product of B and $U_{M}^{⊥}$ is zero space, which satisfies $BU_{Sa}=U_{Sa},BU_{M}^{⊥}=O$.

The received signal is preprocessed by eigen-oblique projection, and the processed signal is:

(25) $$y\left(k\right)=Bx\left(k\right)$$

By the eigen-oblique projection matrix’s nature, the mainlobe interference component has been eliminated, the loss of the desired signal has been reduced, and the sidelobe interference has been preserved.

### 3.2. The Reconstruction of SINCM

SINCM of the entire sidelobe area is as follows:

(26) $$\begin{array}{cc} R_{xS} & =\sum_{i=1}^{S}R_{xSi}\\ & =\sum_{i=1}^{S}\sum_{j=1}^{\Theta_{Si}/\Delta \theta }\frac{a\left(\theta_{j}\right)a^{H}\left(\theta_{j}\right)}{a^{H}\left(\theta_{j}\right)R_{x}{}^{-1}a\left(\theta_{j}\right)}\Delta \theta \end{array}$$

Since the angle area of sidelobe interference is small, noise in the covariance matrix is negligible.

In the proposed method, the noise power can be calculated by:

(27) $$\sigma_{n}{}^{2}=\frac{\sum_{n=N-M-S+2}^{N}{\tilde{\lambda }}_{n}}{N-M-S-1}$$

${\tilde{\lambda }}_{N-M-S-1},\cdots ,{\tilde{\lambda }}_{N}$ represents the noise eigenvalue.

The reconstructed SINCM is expressed as follows:

(28) $${\tilde{R}}_{x}=R_{xS}+\sigma_{n}{}^{2}I$$

### 3.3. Adaptive Weight Vector Calculation

Based on the previous discussion, replacing $R_{x}$ with ${\tilde{R}}_{x}$ to obtain the adaptive weight vector:

(29) $$W=\frac{{\tilde{R}}_{x}{}^{-1}a\left(\theta_{0}\right)}{a^{H}\left(\theta_{0}\right){\tilde{R}}_{x}{}^{-1}a\left(\theta_{0}\right)}$$

The ULA output data is as follows:

(30) $$z\left(k\right)=W^{H}Bx\left(k\right)$$

### 3.4. Summary of Proposed Method

Finally, the steps of the proposed algorithm can be summarized as Algorithm 1:

**Algorithm 1** Multiple mainlobe interferences suppression algorithm**Input:** received data x**Output:** output data z1: Calculate the MUSIC spectrum $P_{\mathrm{Music}}(\theta )$ of the received data x by Equation (11);2: Reconstruct the covariance matrices $R_{xMi}$ and $R_{xSi}$ respectively by Equations (13) and (21);3: Process $R_{xMi}$ and $R_{xSi}$ with eigen-decomposition, construct subspaces $U_{M}^{⊥}$ and $U_{Sa}$ by Equations (16), (17) and (23), calculate eigen-oblique projection matrix B according to Equation (24);4: Reconstruct the SINCM ${\tilde{R}}_{x}$ by Equations (26) and (28);5: Calculate the beamformer adaptive weight vector W by Equation (29), and calculate the output data z by Equation (30).

## 4. Simulation Results

Consider a ULA with 64 omnidirectional antennas. The elements gap is half wavelength. The desired signal is incident from 0° with the signal-to-noise ratio (SNR) 0 dB. Two mainlobe interferences are incident from −2° and 5° with the same interference-to-noise ratio (INR) of 5 dB. Two sidelobe interferences are incident from −15° and 10° with INR 25 dB and 20 dB, respectively. The interference signal is independent of the desired signal. The noise is additive white Gaussian noise. To analyze the experimental results, we conducted 200 Monte Carlo simulations. The proposed method is compared to SMI, EMP-SC [14], EMP-CMR [2], EMP-CMYR [17], EMP-CMIR [16], EMP-IAA [19], and EMP-CS [20]. It is worth noting that the received data contains no desired signal in EMP-SC and EMP-CMR.

### 4.1. Comparison of Beam Pattern

**Figure 2:**
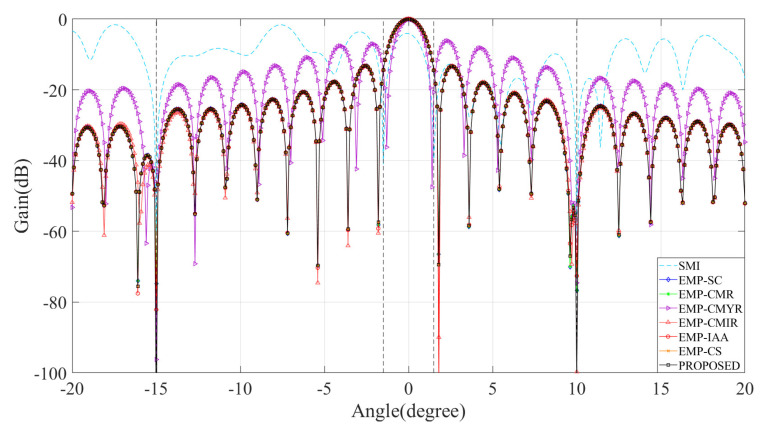
Comparison of beam pattern.

Figure 2 shows beam patterns of the seven methods in 200 Monte Carlo simulations. It can be seen that mainlobe interference affects the beam pattern of the SMI method, forming a null inside the mainlobe that causes mainlobe distortion. In contrast, other methods obtain an ideal mainlobe beam and form a deep null at the sidelobe interference, which effectively solves the mainlobe distortion problem and adaptively eliminates the sidelobe interference.

### 4.2. Comparison of Array Output Data

**Figure 3:**
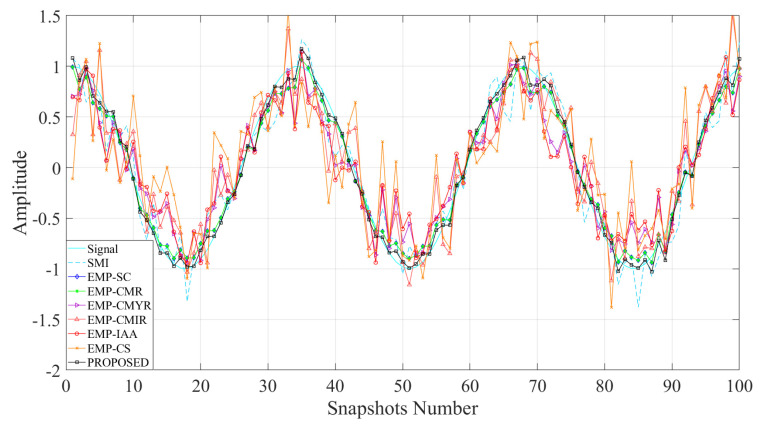
Comparison of array output data.

Figure 3 compares each method’s output data to the desired signal. The figure shows that the desired signal output of EMP-CMIR is seriously distorted in the case of multiple mainlobe interferences. On the one hand, the mainlobe interference eigenvector is mismatched with the corresponding steering vector. On the other hand, the sidelobe interference steering vector after preprocessing has changed. The proposed method solves these two problems, thus improving the interference suppression capability.

In Table 1, we compare the related coefficients between each method’s output data and the desired signal. The correlation coefficient is defined as follows:

(31) $$\rho_{xz}=\frac{\sum_{k=1}^{K}x\left(k\right)z^{*}\left(k\right)}{{\left[\sum_{k=1}^{K}{\left|x\left(k\right)\right|}^{2}\sum_{k=1}^{K}{\left|z\left(k\right)\right|}^{2}\right]}^{1/2}}$$

The correlation coefficient is used to measure the correlation between the desired signal x and the output signal z. Table 1 shows that EMP-SC, EMP-CMR, and the proposed method have the highest correlation coefficient, which means the output data of the three methods are closer to the desired signal. However, the output data of the EMP-CMYR, EMP-CMIR, EMP-IAA, and EMP-CS are quite different from the desired signal. In the multiple mainlobe interference scene, the EMP-CMYR method reconstructs the covariance matrix of the whole mainlobe angle area, which causes a mismatch between the mainlobe interference eigenvector and steering vector. The EMP-CMIR method changes the spatial characteristics of sidelobe interference after eigen-decomposition, which leads to the weakening of its ability to suppress sidelobe interference. The IAA estimation adopted by the EMP-IAA method can process coherent signals, but the spatial resolution is low. It is unable to effectively distinguish multiple mainlobe interferences, which reduces the mainlobe interference suppression effect. The EMP-CS method reconstructs the interference covariance matrix in a sparse manner. However, this method uses traditional methods to estimate source DOA with a fixed upper limit of spatial resolution. Moreover, this method cannot solve the problem of mismatch of interference angle grid estimation.

### 4.3. Analysis of the Impact of Input SNR on Output SINR

**Figure 4:**
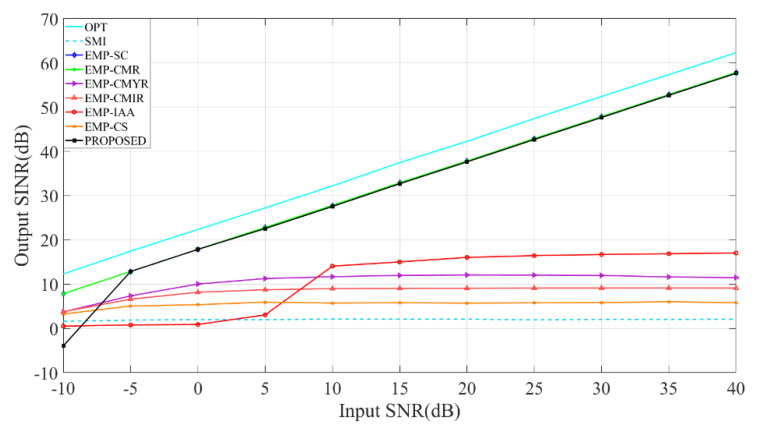
Analysis of the impact of Input SNR on Output SINR.

Figure 4 compares each method’s output SINR when the input SNR increases from −10 dB to 40 dB. It should be pointed out that in the simulations of EMP-SC and EMP-CMR methods, there is no desired signal. Simulation results show that the proposed method’s output SINR is similar to that of EMP-SC and EMP-CMR. When the input SNR is −5 dB, the proposed method can obtain high output SINR, while other methods have poor performance in the case of multiple mainlobe interferences. Compared with EMP-SC and EMP-CMR, since the proposed method avoids the influence of the desired signal in the received data through eigen-subspace reconstruction, it is more practical.

### 4.4. Analysis of the Impact of Snapshots Number on Output SINR

**Figure 5:**
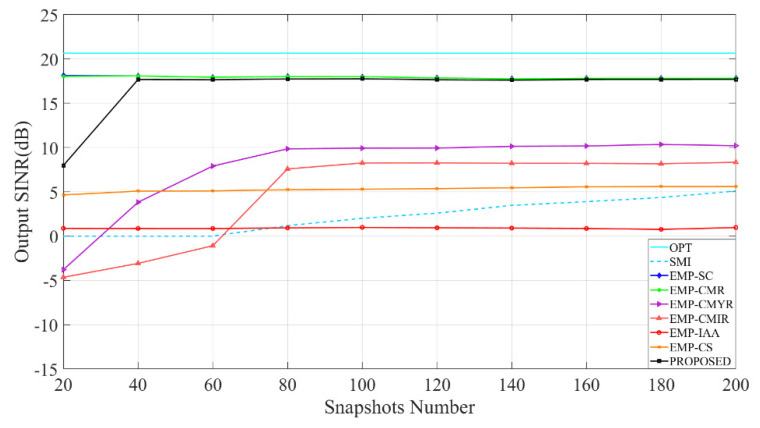
Analysis of the impact of snapshots number on output SINR.

Figure 5 compares each method’s output SINR; when the snapshots number increases from 20 to 200, it can be seen that EMP-SC, EMP-CMR, and the proposed method can obtain higher output SINR. When the snapshots number is 40, the proposed method’s output SINR has approached the optimum. It shows that the method converges faster than other methods.

### 4.5. Analysis of the Impact of Input SNR on ISR

**Figure 6:**
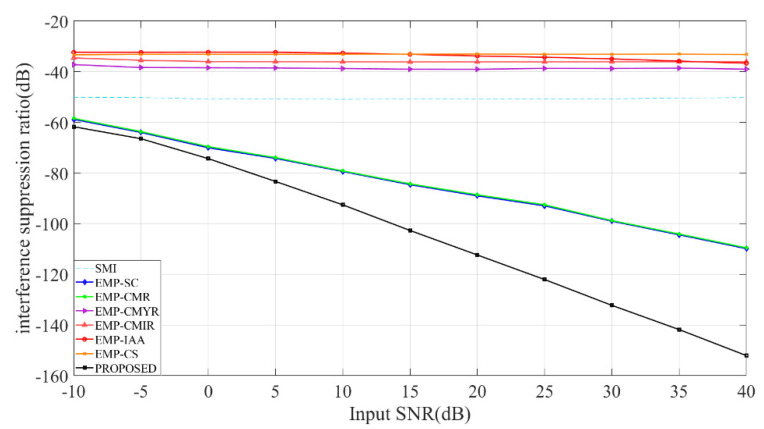
Analysis of the impact of input SNR on ISR.

Figure 6 compares each method’s ISR when the input SNR increases from −10 dB to 40 dB. Simulation results show that the ISR of EMP-CMYR, EMP-CMIR, EMP-IAA, and EMP-CS methods basically does not change with the input SNR. The reason is that the interference suppression performance of these four methods is mainly affected by the interference DOA accuracy. The proposed method can obtain the lowest ISR, which means it has the best overall interference suppression effect.

## 5. Conclusions

In this paper, the authors introduce a multiple mainlobe interferences suppression method based on eigen-subspace and eigen-oblique projection. Compared to existing methods, the proposed method improves interference subspace reconstruction. The accuracy of the mainlobe interference eigenvector obtained is better than that of similar methods, so the eigen-oblique projection matrix has the best performance in suppressing mainlobe interference. At the same time, the eigen-oblique projection retains the spatial characteristics of sidelobe interference, which means the adaptive weight vector can suppress sidelobe interference well. The simulation results indicate that this method can suppress multiple mainlobe interferences and obtain higher ISR than other methods.

## Figures and Tables

**Table 1:** Comparison of related coefficients.

Method	Related Coefficient
SMI	0.9602
EMP-SC	0.9677
EMP-CMR	0.9677
EMP-CMYR	0.8188
EMP-CMIR	0.3228
EMP-IAA	0.6030
EMP-CS	0.8660
PROPOSED	0.9680

## Data Availability

The computer program data used to support the findings of this study are available from the corresponding author upon request.
